# Vitamin E Intake and Risk of Prostate Cancer: A Meta-Analysis

**DOI:** 10.3390/nu15010014

**Published:** 2022-12-21

**Authors:** Wei Qi Loh, Jiyoung Youn, Wei Jie Seow

**Affiliations:** 1 Saw Swee Hock School of Public Health, National University of Singapore and National University Health System, 12 Science Drive 2, #10-01, Singapore 117549, Singapore; 2 Department of Food and Nutrition, College of Human Ecology, Seoul National University, Seoul 08826, Republic of Korea

**Keywords:** vitamin E, prostate cancer, nutritional epidemiology, meta-analysis, dietary, supplements

## Abstract

Vitamin E is a group of antioxidative tocopherols and tocotrienols that play a potential role in chemoprevention. Studies investigating the association between vitamin E and prostate cancer risk have been conflicting. We identified observational and interventional studies examining the association between vitamin E intake and prostate cancer risk from PubMed, EMBASE and the Cochrane Library. A random-effects model was used to perform a meta-analysis and estimate relative risks (RRs) and the corresponding 95% confidence intervals (CIs) of prostate cancer risk according to vitamin E intake. Subgroup analyses were conducted by study design, sample size, study population characteristics, geographical region, and dose of vitamin E intake. The association between dietary (RR = 0.97; 95% CI = 0.92–1.02) and supplemental (RR = 0.99; 95% CI = 0.94–1.04) vitamin E intake on prostate cancer risk was non-significant. In subgroup analyses, supplemental vitamin E was significantly associated with reduced prostate cancer risk in studies in Europe (RR = 0.81, 95% CI = 0.69–0.97). Overall, this meta-analysis demonstrates little evidence for a beneficial effect of vitamin E intake on prostate cancer risk but suggests that there may be some conditions in which supplements could confer a protective effect on prostate cancer risk.

## 1. Introduction

Prostate cancer is characterised by uncontrolled cell growth within the prostate, which is a small muscular gland located below the bladder in men. In more than half of the countries in the world, prostate cancer is the most frequently diagnosed cancer among men [1]. Globally, there were an estimated 1,414,259 newly diagnosed cases in 2020 [1]. However, the etiology of prostate cancer has remained poorly understood compared to other common cancers [2,3].

Vitamin E is a group of antioxidant fat-soluble micronutrients (α-, γ-, δ-, β-tocotrienol and α-, γ-, δ-, β-tocopherol) that is found in high amounts (>10 mg per 100 mL or 100 mg) in nuts, seeds and vegetable oil [4]. It has been reported that consuming 1.5 ounces of almonds or hazelnuts may provide up to approximately 75% of the recommended intake of 15 mg/day. Vitamin E has been identified as a potential chemopreventive agent due to its radical-scavenging antioxidative effects [5,6]. Both tocopherols and tocotrienols have demonstrated effectiveness in the growth inhibition of prostate cancer cells in in vitro studies [7,8]. Many studies, including large-scale randomized controlled trials (RCTs), have attempted to investigate the potential chemopreventive effects of supplemental vitamin E in prostate cancer, but findings have been inconsistent [9,10,11,12,13,14].

Previously, a meta-analysis of RCTs using vitamin E-containing interventions estimated a significant protective effect for vitamin E on prostate cancer risk [15]. However, this meta-analysis only included five studies and its findings were largely driven by a single study, the Alpha-Tocopherol Beta-Carotene (ATBC) trial. In another meta-analysis of both observational and interventional studies, no associations were found between the use of vitamin E supplements and prostate cancer risk [16]. Meanwhile, no meta-analysis has been conducted to date on the association between dietary vitamin E and prostate cancer risk. The effectiveness of dietary and supplemental vitamin E intake may differ, given the differences in stereoisomerism between natural and synthetic forms [17]. Additionally, vitamin E is a frequently consumed supplement, particularly in Western populations [18,19]. Therefore, our study aimed to provide updated evidence on the respective associations between dietary, supplemental, or total vitamin E intake with prostate cancer risk.

## 2. Materials and Methods

The protocol was developed in accordance with the Preferred Reporting Items for Systematic Reviews and Meta-Analyses (PRISMA-P) statement [20].

### 2.1. Search Strategy

A search of the electronic databases PUBMED, EMBASE, and the Cochrane Library for references published before 30 November 2021 was undertaken. We used a combination of standard controlled vocabulary (MeSH and Emtree) and keywords for (1) vitamin E and (2) prostate cancer to identify relevant references that studied the association between vitamin E and prostate cancer risk. The search strings used for all three databases are presented in Supplementary Table S1. For each database, all references identified with the final search terms were downloaded into Endnote X9 software (Version 3.3, Clarivate Analytics, London, UK). We also searched through the reference lists of studies that fulfilled the eligibility criteria in screening to identify additional references.

### 2.2. Eligibility Criteria

We included RCTs and observational studies (case-control studies, cohort studies, case-cohort studies, nested case-control studies) that studied the association between vitamin intake (including any of its eight isoforms: α-, β-, γ-, and δ-tocopherol as well as α-, β-, γ-, and δ-tocotrienol) and prostate cancer risk in our meta-analysis. RCTs evaluating the use of a multi-vitamin supplement were eligible if vitamin E was described as a component. Studies had to have enrolled adult male participants over 40 years of age and presented risk estimates of prostate cancer (risk ratio, hazard ratio, odds ratio) as well as the respective 95% CI according to categories of dietary and/or supplemental vitamin E intake.

If multiple studies used data from the same population, the study with the longest follow-up time was included in this meta-analysis. Included studies had to be written in English and published in peer-reviewed journals.

### 2.3. Study Selection and Data Extraction

Two reviewers, LWQ and YJY, independently screened all references downloaded from the three databases. The following data items were extracted from the included studies: publication information (article title, author details, journal, year of study, location of study), study population (sample size, study design, demographics such as age and ethnicity), exposure or intervention of vitamin E intake (type of vitamin E intake [i.e., dietary, supplemental or total]), prostate cancer risk estimates of highest versus lowest category of vitamin E intake (risk ratios, rate ratios, odds ratios or hazards ratios, and their respective 95% CI), as well as any covariates that were adjusted for in the models. When results from multiple statistical models were presented, risk estimates and the respective 95% CI were extracted from the model that adjusted for more covariates. Data extraction was completed through an electronic form developed in Microsoft Excel. Any inconsistencies between the two reviewers were resolved through discussion and consensus.

### 2.4. Quality Assessment

Two reviewers, LWQ and YJY, evaluated the risk of bias of individual studies independently. RCTs were assessed using the Revised Cochrane Risk-of-Bias (RoB 2) tool, which consists of five domains: (1) bias arising from the randomization process, (2) bias due to deviations from intended interventions, (3) bias due to missing outcome data, (4) bias in the measurement of the outcomes, and (5) bias in the selection of the reported result [21]. The risk levels were classified as ‘low risk of bias’, ‘some concerns’ and ‘high risk of bias’ according to the signalling questions provided.

Observational studies were assessed using the Cochrane tool Risk of Bias In Non-randomised Studies of Interventions (ROBINS-I) [22]. This tool evaluated the internal validity of individual studies across seven domains, comprising: (1) bias due to confounding, (2) selection bias, (3) bias in classification of interventions, (4) bias due to departure from intended interventions, (5) bias due to missing data, (6) bias in measurement of outcomes, and (7) bias in selection of reporting of results. Each domain was categorized to be at “low risk”, “moderate risk”, “serious risk” or “critical risk” of bias according to the signalling questions provided. Any disagreements were resolved through discussion among the two reviewers.

### 2.5. Statistical Analysis

Using data extracted from the included studies, pooled risk estimates and respective 95% CIs for prostate cancer risk according to vitamin E intake (highest category versus lowest category) were computed. We chose to implement the random effects model in this meta-analysis as the studies were clinically heterogeneous (e.g., different study designs, doses of vitamin E intake), and were likely to estimate different underlying true effects. The restricted maximum likelihood method was used to estimate the heterogeneity variance. We examined and quantified the statistical heterogeneity of the study results using a Cochrane Q test and I^2^ values. Synthesis of the overall estimate was done with careful consideration of the heterogeneity, making sure that studies were only combined under appropriate heterogeneity levels (<70%). Subgroup analyses according to study design, sample size, study population characteristics, geographical region and dose of vitamin E intake were conducted to determine if pooled risk estimates differed across these characteristics. To assess the robustness of our findings, we performed sensitivity analyses by excluding one study at a time to examine the magnitude of influence each study had on the pooled risk estimates. We also carried out further sensitivity analyses by excluding studies with a high or serious risk of bias, as determined by the RoB2 and ROBINS-I tools, as well as limiting studies to those that had adjusted for important confounders (i.e., energy intake and family history of prostate cancer). We also attempted to estimate the trend of relative risk estimates across increasing categories of vitamin E intake through a dose-response meta-analysis using the ‘dosres’ package in R [23,24,25].

We examined small study effects, including potential publication bias, by carrying out a funnel plot analysis and assessing asymmetry using an Egger’s regression test. The trim-and-fill method was applied to calculate an adjusted effect size. All analyses were done using the ‘metafor’ package in R (R Version 4.0, R Core Team, Vienna, Austria) [26]. All *p* values were two-sided, with a value of <0.05 being considered as statistically significant.

## 3. Results

### 3.1. Characteristics of Studies Selected

**Figure 1:**
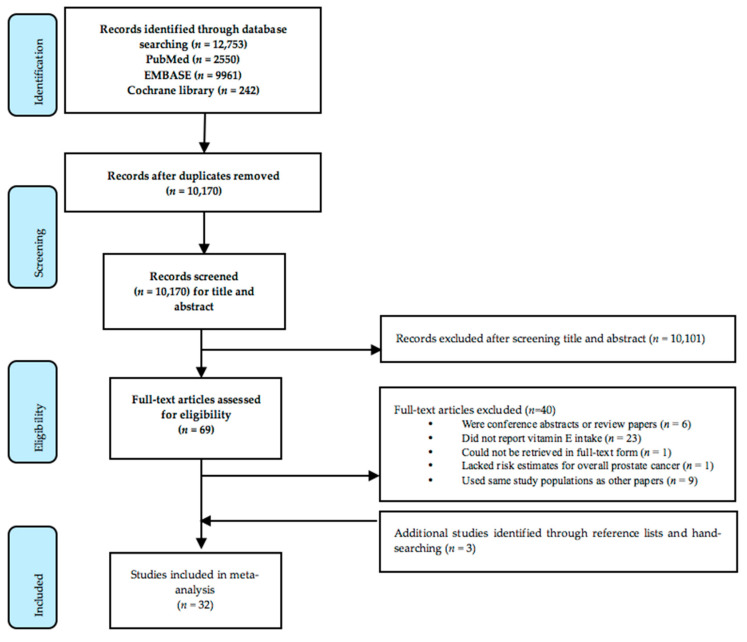
PRISMA flowchart outlining the study selection and data extraction process.

Details of the study selection process are presented in a PRISMA flowchart (Figure 1). A total of 12,753 potentially relevant articles were identified from the databases of PubMed, EMBASE and the Cochrane Library. A total of 32 studies were included in the meta-analysis after screening.

Table 1 presents the characteristics of 32 studies included in this meta-analysis evaluating the association between vitamin E intake and overall prostate cancer risk. The age of the participants ranged from 35 to 89 years. The majority of the studies were conducted in the United States (12 studies), Europe (11 studies) and Canada (five studies). The remaining studies took place across several countries (two studies), or were conducted in Australia (one study) and Uruguay (one study).

We identified 19 studies that investigated associations between dietary intake of vitamin E and prostate cancer risk. This included 11 case-control studies, six prospective cohort studies, one case-cohort study and one nested case-control study. In total, the analysis comprised 516,753 participants, in which 27,141 cases of prostate cancer were identified.

Meanwhile, we identified a total of 18 studies that evaluated the supplemental intake of vitamin E and prostate cancer risk, comprising six RCTs, nine prospective cohort studies, two case-control studies and one case-cohort study. Here, the analysis consisted of 686,348 non-overlapping participants, among which 31,274 cases of prostate cancer had been identified.

There were five studies evaluating the association between total (both diet and supplemental) intake of vitamin E and prostate cancer risk. This comprised one case-control study, three prospective cohort studies and one case-cohort study. In total, the analysis included 94,421 non-overlapping participants, in which 5122 cases of prostate cancer had been identified.

### 3.2. Overall Analysis of Vitamin E Intake and Prostate Cancer

**Figure 2:**
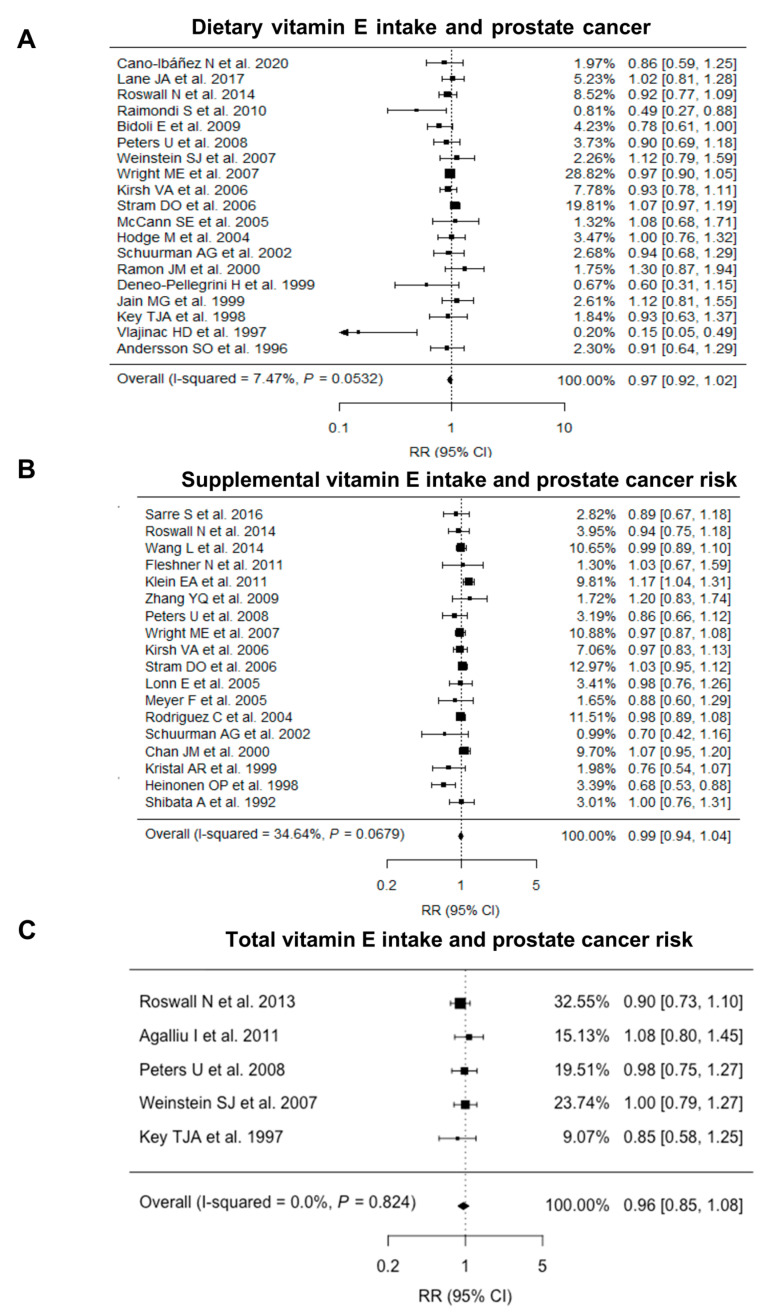
Forest plot for associations between (**A**) dietary, (**B**) supplemental and (**C**) total vitamin E intake and prostate cancer risk. CI: Confidence interval; RR: Relative risk.

The pooled relative risk estimates of prostate cancer risk were 0.97 (95% CI = 0.92–1.02, I^2^ = 7.47%; Figure 2A) for dietary vitamin E intake and 0.99 (95% CI = 0.94–1.04, I2 = 34.64%; Figure 2B) for supplemental vitamin E intake. There was no evidence of an association between total (dietary and supplemental) vitamin E intake and prostate cancer risk (RR = 0.96, 95% CI = 0.85–1.08, I^2^ = 0.00%; Figure 2C). Overall, the statistical heterogeneity for the three pooled relative risk estimates were either low (I^2^ < 25%) or moderate (25% ≤ I^2^ < 50%).

### 3.3. Small-Study Effects and Quality Analysis

**Figure 3:**
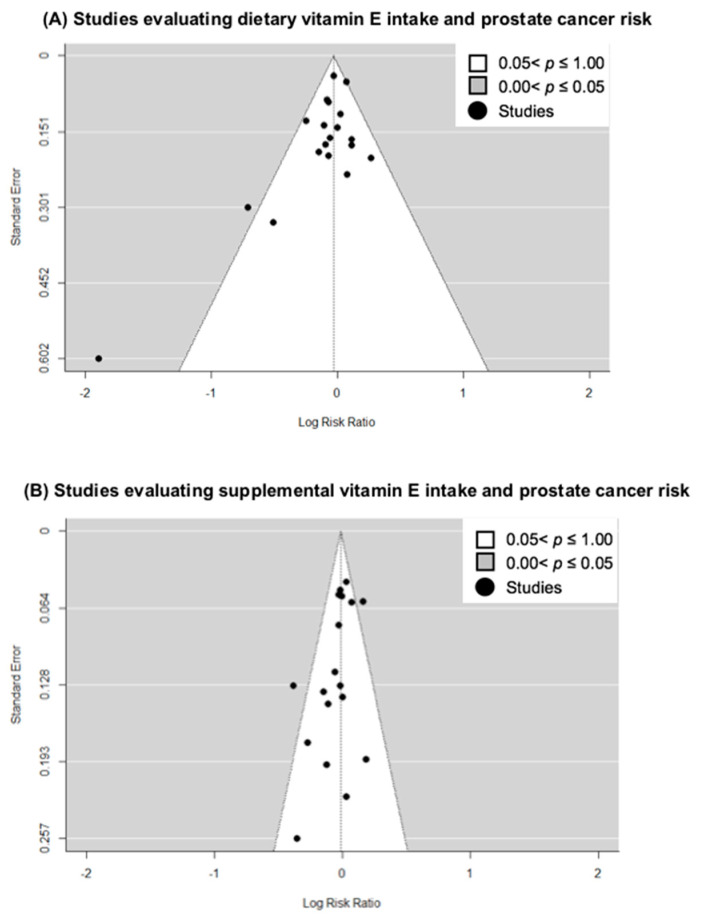
Funnel-plot analyses of studies included in meta-analysis for (**A**) dietary vitamin E intake and (**B**) supplemental vitamin E intake.

To assess the presence of small-study effects, we carried out funnel-plot analyses of the included studies on dietary (Figure 3A) and supplemental vitamin E intake (Figure 3B). Using Egger’s regression test for funnel plot asymmetry, we found borderline significant evidence for small study effects, including publication bias, for the studies on dietary intake (*p* value = 0.060) and supplemental intake (*p* value = 0.048) and prostate cancer risk. Applying the trim-and-fill method did not drastically alter the risk estimates (RR = 0.98, 95% CI = 0.93–1.03 for dietary intake; RR = 1.01, 95% CI = 0.95–1.08 for supplemental intake). We did not carry out an assessment of small-study effects for studies on total vitamin E intake as the number of studies with this variable were limited.

**Figure 4:**
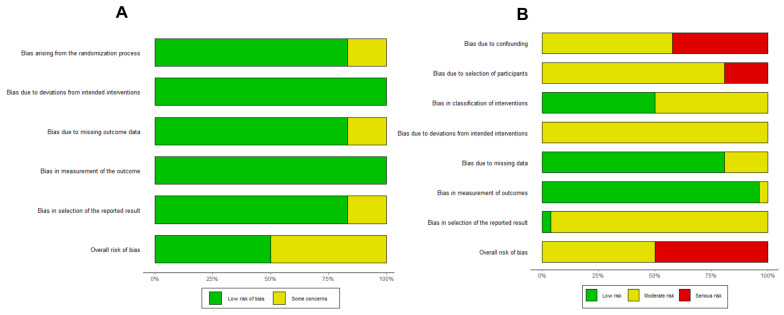
Summary bar plots assessing the risk of bias in (**A**) randomized controlled trials and (**B**) observational studies using the RoB2 and ROBINS-I tools, respectively.

The overall risk of bias for RCTs and observational studies are presented in Figure 4A,B. Out of the six RCTs, three were rated as ‘low risk’ of bias, while the remaining three were assessed to have ‘some concerns’ of bias. All observational studies included in our analysis were assessed to be at either ‘moderate’ risk of bias (13 out of 26 studies), or at ‘serious’ risk of bias (13 out of 26 studies). Potential confounding and/or selection bias were the most concerning issues that led to ‘serious’ risk of bias.

### 3.4. Sensitivity Analyses

During the leave-one-out sensitivity analysis, pooled relative risk estimates remained largely similar to original estimates for dietary/supplemental intake of vitamin E and prostate cancer risk. However, when excluding the prospective cohort study by Stram et al., the inverse association between dietary vitamin E intake and prostate cancer risk attained borderline significance (RR = 0.95; 95% CI = 0.90–1.00; I^2^ = 0.04%). Further sensitivity analyses by excluding observational studies at ‘serious’ risk of bias or RCTs with ‘some concerns’ of bias did not alter our findings for dietary vitamin E (RR: 0.97; 95% CI = 0.91–1.04; I^2^ = 18.98%) or supplemental vitamin E (RR = 0.99; 95% CI = 0.95–1.03; I^2^ = 0.00%). In addition, restricting studies to those that had adjusted for energy intake (dietary vitamin E: RR = 0.98; 95% CI = 0.92–1.04; I^2^ = 10.13%; supplemental vitamin E: RR = 0.95; 95% CI = 0.83–1.09; I^2^ = 0.00%) or family history of prostate cancer (dietary vitamin E: RR = 0.95, 95% CI = 0.88–1.03; I^2^ = 27.77%); supplemental vitamin E: RR = 1.00; 95% CI = 0.95–1.04; I^2^ = 0.00%) did not alter our findings. There was limited evidence of a dose-response relationship for dietary or supplemental vitamin E intake and prostate cancer risk. Sensitivity analysis for studies on total vitamin E intake were not conducted, as the number of studies on this variable were limited.

### 3.5. Subgroup Analyses

Results of the subgroup analyses are summarised in Table 2. For dietary vitamin E, there remained no significant associations between intake and prostate cancer risk in the subgroup analyses according to study design, sample size, geographical region, and amount of vitamin E intake. There was a significant subgroup difference for studies conducted in Europe as compared to North America (*p* value for subgroup differences = 0.036).

For supplemental vitamin E intake, the pooled risk estimates in studies of European populations (RR = 0.81; 95% CI = 0.69–0.97; I^2^ = 33.56%) and North American populations (RR = 1.01; 95% CI = 0.97–1.06; I^2^ = 18.76%) were significantly different (*p* value for subgroup differences = 0.020). However, study design, study type, sample size, dose of supplements used, as well as the presence of underlying conditions in participants of RCTs, did not appear to influence estimates of prostate cancer risk with supplemental vitamin E intake.

## 4. Discussion

In this meta-analysis, we quantitatively estimated the associations between dietary and supplemental vitamin E and prostate cancer risk by pooling estimates in observational and interventional studies published to date.

Although the risk estimates (RR = 0.97; 95% CI = 0.92–1.02; I^2^ = 7.47%) were suggestive of a slight reduction in prostate cancer risk for high dietary intake of vitamin E, there was no statistical evidence for a benefit. This implies that a high dietary intake of vitamin E may not account for the inverse associations between serum/plasma vitamin E (in particular, α-tocopherol) and prostate cancer risk that have been reported in the literature [54,55]. Apart from dietary intake, serum/plasma concentrations of α-tocopherol can be influenced by other factors including genetics, use of supplements, seasonality, ethnicity, and location of residence [56,57,58,59,60]. In a nested case-control study of the Prostate, Lung, Colorectal, and Ovarian Cancer Screening Trial, minor alleles on an single nucleotide polymorphism rs964184 located near the apolipoprotein 5 gene, known to be involved in vitamin E transport and metabolism, were significantly inversely associated with prostate cancer risk [61].

Supplemental intake of vitamin E was not associated with prostate cancer risk in this study. This null association is consistent with findings from previous reviews [15,16,62]. However, we found statistically significant subgroup differences between studies conducted in Europe and North America regarding the effect of vitamin E. Further, a significant inverse association was observed between supplemental vitamin E and prostate cancer risk when combining studies in Europe. One possible explanation for this observation is the difference in predominant forms of dietary vitamin E in these regions. Vitamin E forms can be found in varying proportions in vegetable oils: α-tocopherol is found in higher amounts in sunflower and olive oil while γ-tocopherol is more common in corn and soybean oil [63,64]. Although α-tocopherol is the predominant dietary form in Europe as well as commercial vitamin E supplements, γ-tocopherol is the predominant form of dietary vitamin E in countries in the North America region, including the United States [65,66]. Importantly, γ-tocopherol acts as a weak competitive substrate to α-tocopherol for binding to hepatic α-tocopherol transfer protein, and the intake of α -tocopherol has been inversely associated with circulating γ -tocopherol levels [59,67,68,69,70,71]. The beneficial effect of supplements (typically α-tocopherol) in North America could have been counterbalanced by the relatively high dietary intake of γ-tocopherol and low dietary intake of α-tocopherol as compared to European counterparts. Future research pertaining to the effects of dietary γ-tocopherol, alongside α-tocopherol metabolism, and prostate cancer risk may be useful in better understanding the association observed.

Additionally, differences in fortification and enrichment practices in North America and Europe may have influenced nutritional adequacy in the populations and result in differential benefits of supplementation [72]. However, we acknowledge that due to the relatively few studies from Europe (four studies), it is possible that the significant inverse association between vitamin E supplements and prostate cancer risk is a spurious finding by chance.

When evaluating total vitamin E intake with prostate cancer risk, we found that there were no significant associations. However, we were also limited by the number of eligible studies (five studies) in evaluating this exposure variable.

One strength of our meta-analysis is that a low level of heterogeneity was observed within the overall analysis. Results obtained by combining studies of different designs may lead to bias resulting from heterogeneity. the I^2^ values for the overall analysis were not substantial (<40%), indicating that the studies included could be reasonably combined. Furthermore, our subgroup analysis by study design suggested that there were no differences in effect detected between studies of various designs.

Secondly, our study took into account both the dietary and supplemental intake of vitamin E, as the effect of these intakes are likely to be interrelated. Past meta-analyses have focused on the use of vitamin E supplements alone and prostate cancer risk, or combined a mixture of studies on either dietary and supplemental intake, without considering that the exposure variables were distinct but also additive [15,16,62].

However, there were also several limitations with our meta-analysis. Firstly, the studies used various assessment methods to measure intake, as well as different cut-off values to categorize intake. These variations introduce heterogeneity and make the pooled risk estimates difficult to interpret. Although a dose-response meta-analysis could have addressed this by enabling one to extract multiple risk estimates across varying doses from the same study, we were limited by the number of studies providing sufficient information (i.e., dose levels) for such analysis. We also acknowledge that the lack of association observed in the meta-analysis could be due to a lack of statistical power or a lack of precision due to the inherent information biases of measuring dietary intake through methods such as food-frequency questionnaires and food diaries. Here, non-differential misclassification may bias the results towards the null.

Secondly, we were unable to factor in the bioavailability of vitamin E in our meta-analysis. The absorption rate of vitamin E, which can range from 20% to 80%, is heavily influenced by other factors such as the food matrix present [17]. We were limited in data in this respect, as most of the included studies do not take bioavailability into account in the estimation of vitamin E intake as a variable.

Furthermore, most studies in the meta-analysis do not differentiate between the subtypes of vitamin E. This may be limited by the challenges faced in separation and quantification of tocopherols and tocotrienols in various food types [17]. As a result, we were unable to evaluate or compare the effect of different vitamin E isoforms on prostate cancer risk. To date, studies have mostly focused on α-tocopherol, and some have quantified other vitamin E isoforms based on α-tocopherol equivalents. This may be driven by the fact that α-tocopherol was thought to possess the highest biological activities compared to other isoforms of vitamin E. [66] Future studies should look to investigating the effects of different vitamin E forms on prostate cancer risk, as there is a difference in prevalent forms between geographical regions, and promising experimental and clinical data have emerged on the bioactivities of tocotrienols [73,74,75,76]. Only one study in our meta-analysis had analysed the intake of tocotrienols as distinct variables [36].

Additionally, the studies included in our meta-analysis tended to overlook fortified sources and cooking oils, which could be major sources of vitamin E when computing intake. This could have led to an underestimate of vitamin E intake and/or non-differential misclassification, which may have biased the results towards the null.

Lastly, as the majority of the studies had been conducted in Western populations in Europe or North America, the findings may face limited generalisability to populations from other regions, such as Africa or Asia.

Overall, our meta-analysis implies that neither vitamin E supplements, nor a high dietary intake of vitamin E, are likely to be significantly useful in a chemopreventive capacity for prostate cancer. However, future studies may benefit from investigating the intake of specific vitamin E forms and prostate cancer risk in various geographical regions.

## Figures and Tables

**Table 1:** Characteristics of the studies included in the meta-analysis.

First Author (Year)	Location	Study Name	Design	Study Population	Participants	Cases	Age, yrs	Study Duration	Years of Follow Up	Type of Vitamin E Intake	Intake Assessment Method	Vitamin E Cut-Offs	Adjustments for Covariates
Cano-Ibáñez N (2020) [27]	Spain	CAPLIFE	Case-control study	Cases: Men diagnosed with prostate cancer at two main university hospitals Controls: Population-based controls	704	402	Mean: 66.7 Range: 40–80	2017–2019	-	Dietary	FFQ	Adequate intake (8.6 mg/d to 300 mg/d) vs. inadequate intake	Age, smoking habits, physical activity level, educational level, alcohol intake, and first-degree family history of prostate cancer.
Lane JA (2017) [28]	UK	-	Nested case-control study	Cases: Men in the Dietary Cohort Consortium Studies diagnosed with prostate cancer Controls: Cohort controls	5245	1717	Mean: 62.8 Range: 50–60	1991–2009	Mean: 6.6–13.3 years	Dietary	Food diary	Quintiles: 7.1 mg/d, 9.0 mg/d, 11.1 mg/d, 14.1 mg/d	Age, BMI, socioeconomic, smoking, and marital status, diabetes, and energy intake.
Sarre S (2016) [29]	Finland	FinsRPC	Prospective cohort study	Men participating in the third round of the FrRSPC without previous diagnosis of prostate cancer	11,795	757	Median: 66.0	2004–2013	Median: 6.6 years	Supplemental	Self-reported use of supplements	Use vs. no use	Age.
Roswall N (2013) [30]	Netherlands	-	Prospective cohort study	Male residents in Denmark	26,865	1571	Median: 56.0 Range: 50–64	1993–2010	Median: 14.3 years	Dietary, Supplemental, Total	FFQ, self-reported use of supplements	Quartiles for dietary: 7.3 mg/d, 9.5 mg/d, 12.0 mg/d; Supplements: 0 mg/d, 4.4 mg/d, 10 mg/d; Total: 8.6 mg/d, 12.0 mg/d, 17.7 mg/d	Intake of the three other micronutrients as well as dietary intake or supplemental intake of vitamin E.
Wang L (2014) [11]	United States	PHS II	RCT	Male physicians aged 50 yrs and above	13,980	1373	Mean: 64.3	1997–2011	-	Supplemental	Intervention	400 IU vs. no use every other day	Age, PHS cohort and randomised assignment
Agalliu I (2011) [31]	Canada	CSDLH	Case-cohort study	Male participants in the CSDLH study recruited from universities in Canada	2525	661	Mean: 68.4	1992–2003	Mean: 4.3 years	Total	FFQ, self-reported use of supplements	Quintile (median value reported): 6.3 mg/d, 8.3 mg/d, 14.6 mg/d, 264.4 mg/d, 462.0 mg/d	Age, race, BMI, exercise activity, and education. Adjusted for energy intake using residual method.
Fleshner N (2011) [13]	Canada	-	RCT	Men with high-grade prostatic interepithelial neoplasia diagnosed within 18 months of random assignment	303	80	Median: 62.8	1999–2004	-	Supplemental	Intervention	400 IU/d vs. no use	-
Klein EA (2011) [12]	United States, Canada, Puerto Rico	SELECT	RCT	Men with prostate- specific antigen concentrations of <4.0 ng/mL	17,433	1149	Median: 62.5	2004–2011	-	Supplemental	Intervention	400 IU/d vs. no use	-
Raimondi S (2010) [32]	Montreal, Canada	-	Case-control study	Cases: Men diagnosed with prostate cancer at major teaching hospitals Controls: Population-based controls identified by random-digit dialing	394	197	Range: 35–84	1989–1993	-	Dietary	FFQ	Quartiles: 5.9 mg/d; 7.4 mg/d; 9.2 mg/d	Family history of prostate cancer, age group, total energy intake, and calcium intake.
Bidoli E (2009) [33]	Italy	-	Case-control study	Cases: Men diagnosed with prostate cancer at teaching and general hospitals Controls: Hospital-based controls	2745	1294	Median: 66 Range: 46–74	1992–2002	-	Dietary	FFQ	Tertiles: 12.3 mg/d, 16.7 mg/d	Age, study center, period of interview, education, body mass index, alcohol intake, smoking habits, family history of prostate cancer and total energy intake.
Peters U (2008) [34]	United States	VITAL	Prospective cohort study	Men living in western Washington State covered by the Surveilance, Epidemiology, and End Results cancer registry	35,242	830	Range: 50–76	2000–2004	Not reported	Dietary, Supplemental, Total	FFQ, self-reported use of supplements	Quartiles for dietary: 8.6 mg/d, 12.2 mg/d, 17.1 mg/d. For supplemental: None, 0–30 IU/d, >30–< 400 IU/d, ≥ 400 IU/d. Categories for total: <14.3 mg/d, 14.3–29.3 mg/d, 29.4–98.0 mg/d, ≥98.1 mg/d	Age, family history of prostate cancer, benign prostatic hyperplasia, income, multivitamin use, and stratified on PSA screening in the 2 years before baseline (yes/no), energy intake.
Zhang YQ (2009) [35]	United States	-	Case-control study	Cases: Men diagnosed with prostate cancer at participating hospitals Controls: Hospital-based controls	4110	1706	Mean: 60.1 Range: 40–79	1976–2006	-	Supplemental	Self-reported use of supplements	Duration of use: 10+ years, 5–9 years, 1–4 years, Never or <1 yr use	Age, years of education, body mass index, current alcohol drinking, current smoking, family history of prostate cancer and use of other vitamin/mineral supplements.
Weinstein SJ (2007) [36]	United States	ATBC	Prospective cohort study within trial	Male smoker residents	29,133	1732	Mean: 57.2 Range: 50–69	1985–2004	Up to 19 years	Dietary, Total	FFQ, self-reported use of supplements	Quintiles for total: 7.06 mg/d. 8.36 mg/d, 10.32 mg/d, 14.72 mg/d; Quintiles for dietary: 6.96 mg/d, 8.13 mg/d, 9.65 mg/d, 13.01 mg/d	Age, trial arm, weight, urban residence, education, intakes of total energy, fat, polyunsaturated fatty acids, vitamin C and lycopene.
Wright ME (2007) [37]	United States	NIH-AARP	Prospective cohort study	Men enrolled in the NIH-AARP Diet and Health study	295,344	10,241	Range: 50–71	1995–2000	Up to 5 years	Dietary, Supplemental	FFQ, self-reported use of supplements	Quintile medians for dietary: 4.8 mg/d, 6.5 mg/d, 7.0 mg/d, 8.0 mg/d, 10.0 mg/d; For supplement: 0 IU/d, >0–99 IU/d, 100–199 IU.d, 200–399 IU/d, 400–799 IU/d. ≥800 IU/d	Age, race, smoking status, education, personal history of diabetes, family history of prostate cancer, body mass index, and dietary intakes of red meat, a-linolenic acid, vitamin C, B carotene intake. Dietary tocopherols were adjusted for energy intake using theresidual method.
Kirsh VA (2006) [38]	United States	PLCO Cancer Screening Trial	Prospective cohort study	Men in the screening arm of the PLCO trial	29,361	1338	Mean: 63.3 Range: 55–74	1993–2001	Mean: 4.2 years	Dietary, Supplemental	FFQ, self-reported use of supplements	Quintiles medians for dietary: 8.6 mg/d, 10.2 mg/d, 11.3 mg/d, 12.6 mg/d, 15.8 mg/d; For supplements: 0 IU/d, >0–30 IU/d, >30–400 IU/d,	Age, total energy, race, study center, family history of prostate cancer, BMI, smoking status, physical activity, total fat intake, red meat intake, history of diabetes, aspirin use, number of screening examinations during follow-up period.
Stram DO (2006) [39]	United States	MEC	Prospective cohort study	Men from a large population-based multiethnic cohort	82,486	3922	Range: 45–75	1993–2001	Up to 7 years	Dietary, Supplemental	FFQ	Quintiles for dietary: 3.9 mg/1000 kcal, 4.5 mg/1000 kcal, 5.1 mg/1000 kcal, 6.0 mg/1000 kcal; For supplements: 0–<33.75 mg/d, ≥33.75 mg/d	Age, ethnicity, BMI, education and family history of prostate cancer. Intake of all foods and nutrients were analysed as nutrient densities.
Lonn E (2005) [14]	Canada, United States, Argentina, Brazil, Mexico and 14 Western European countries	HOPE and HOPE-TOO	RCT	Male patients at high risk for cardiovascular events	6996	235	Mean: 66.0	1993–1999; 1999–2003	-	Supplemental	Intervention	400 IU/d vs. 0 IU.d	-
McCann SE (2005) [40]	United States	WNYDS	Case-control study	Cases: Men diagnosed with prostate cancer from major hospitals Controls: Population-based controls	971	433	Mean: 69.5	1986–1991	-	Dietary	FFQ	Quartile range: <7 mg/d. 7–9 mg/d, 9–11 mg/d, >11 mg/d	Age, education, BMI, cigarette smoking status, total energy, vegetable intake.
Meyer F (2005) [10]	Canada	SU.VI.MAX	RCT	Healthy male volunteers	5034	103	Mean: 51.3 Range: 45–60	1994–2002	-	Supplemental	Intervention	30 mg/day vs. no use	-
Hodge M (2004) [41]	Australia	-	Case-control study	Cases: Australian male residents with prostate cancer Controls: Population-based controls	1763	858	Range: < 70	1994–1997	-	Dietary	FFQ	Quintile range: <6.9 mg/d, 6.9–8.0 mg/d, 8.1–9.0 mg/d. 9.1–10.3 mg/d, ≥10.4 mg/d	State, age group, year, country of birth, socio-economic group, family history of prostate cancer. Nutrient adjusted for energy intake by residual method.
Rodriguez C (2004) [42]	United States	CPS-II	Prospective cohort study	Men selected from the CPS-II Nutrition Cohort	72,704	4281	Range: 50–74	1992–1999	Not reported	Supplemental	FFQ	None, 1–31 IU/d, 32–≤400 IU/d, ≥400 IU/d	Age, race, smoking status, BMI, education, energy adjusted calcium, total fat, lycopene intake, total calorie intake, family history of prostate cancer, and PSA history.
Schuurman (2002) [43]	Netherlands	NLCS	Case-cohort study	Men from the study population in NLCS	2167	642	Mean: 62.1 Range: 55–69	1986–1992	Up to 6.3 years	Dietary, Supplemental	FFQ, self-reported use of supplements	Quintile medians: 7.1 mg/d, 10.4 mg/d, 13.5 mg/d, 17.3 mg/d, 23.6 mg/d	Age, family history of prostate cancer, socioeconomic status, and alcohol from white or fortified wine.
Ramon JM (2000) [44]	Spain	-	Case-control study	Case: Men diagnosed with prostate cancer in hospital records Controls: Hospital and population-based controls	651	217	-	1994–1998	-	Dietary	FFQ	Quartile medians: 6.1 mg/d, 7.6 mg/d, 9.9 mg/d, 12.8 mg/d	Age, residence, calories, family history and BMI.
Chan JM (2000) [45]	United States	HPFS	Prospective cohort study	Male health professionals	47,780	1896	Mean: 54.6 Range: 40–75	1986–1996	Not reported	Supplemental	FFQ and self-reported use of supplements	0 IU/d, 0.1–15.0 IU/d, 15.1–99.9 IU/d, ≥100 IU/d	Age, period, family history of prostate cancer, vasectomy, smoking, quintiles of BMI, BMI at age 21, physical activity, quintiles of total calories, calcium, lycopene, fructose, and fat intake per day.
Deneo-Pellegrini H (1999) [46]	Uruguay	-	Case-control study	Case: Men diagnosed with prostate cancer admittted to major hospitals Controls: Hospital-based controls	408	175	Range: 40–89	1994–1997	-	Dietary	FFQ	Quartile ranges: ≤5.0 mg/d, 5.1–6.0 mg/d, 6.1–7.8 mg/, ≥7.9 mg/d	Age, residence, urban/rural, family history of prostate cancer, BMI, total energy intake.
Jain MG (1999) [47]	Canada	-	Case-control study	Cases: Men recently diagnosed with prostate cancer identified by hospital admission offices or cancer registries Controls: Population-based controls	1253	617	Mean: 69.9	1989–1993	-	Dietary	FFQ	Quartile ranges: <17.17 mg/d, 17.17–25.30 mg/d, 25.31 mg/d, 37.25 mg/d, ≥37.25 mg/d	Age, log total energy intake, vasectomy, marital status, ever smoke study area, BMI, education, ever-used multivitamin supplements, area of study, log-amounts for grains, fruits, vegetables, total plants, total carotenoids, folic acid, dietary fibre, conjugated linoleic acid, vitamin E, vitamin C, retinol, total fat, and linolenic acid.
Kristal AR (1999) [48]	United States	-	Case-control study	Men diagnosed with prostate cancer, identified from the Seattle-Puget Sound SEER cancer registry Controls: Population-based controls	1363	697	Range: 40–64	1993–1996	-	Supplemental	Self-reported use of supplements	Frequency of use: 0/week, <1/week, 1–6/week, ≥7/week	Age, race, education, energy, family history of prostate cancer, body mass index, number of PSA tests in previous 5 years, dietary fat intake.
Heinonen OP (1998) [9]	Finland	ATBC	RCT	Male smokers residents	29,133	246	Mean 57.1 Range: 50–69	1985–1993	-	Supplemental	Intervention	50 mg/d vs. no use	-
Key TJA (1997) [49]	UK	-	Case-control study	Cases: Men diagnosed with prostate cancer based on hospital registry records Controls: Patients of the general pracitioners for cases	656	328	Mean: 68.1	1990–1994	-	Dietary, Total	FFQ	Tertile ranges for dietary: <9.59 mg/d, 9.59–16.33 mg/d, ≥16.34 mg/d; Tertile ranges for total: <9.94 mg/d, 9.94–17.87 mg/d, ≥17.88 mg/d	Energy.
Vlajinac HD (1997) [50]	Serbia	-	Case-control study	Cases: Patients diagnosed with prostate cancer Controls: Hospital-based controls	303	101	Mean: 71.2	1990–1994	-	Dietary	FFQ	Tertiles; no cut-off values reported	Energy, protein, fat-total, saturated fatty acids, carbohydrate, sugar, fibre, retinol, retinol equivalent, folic acid, vitamin B12, sodium, potassium, calcium, phosphorous magnesium and iron.
Andersson SO (1996) [51]	Sweden	-	Case-control study	Cases: Male residents in Sweden diagnosed with prostate cancer, identified through hospital records Controls: Population-based controls	1062	526	Mean: 70.6	1989–1994	-	Dietary	FFQ	Quartiles: 4.5 mg/d, 5.7 mg/d, 7.3 mg/d	Age and energy adjusted, based on nutrient residuals and energy in quartiles.
Shibata A (1992) [52]	United States	-	Prospective cohort study	Male residents of a retirement community	4252	207	Mean: 74.9	1981–1989	Up to 8 years	Supplemental	Self-reported use of supplements	Use vs. no use	Age and smoking habits.

ATBC: Alpha-tocopherol, Beta-Carotene Cancer Prevention Study; BMI: Body mass index; CAPLIFE: Prostate cancer lifestyles study; CPS-II: Cancer Prevention Study II; CSDLH: Canadian Study of Diet, Lifestyle, and Health; FFQ: Food frequency questionnaire; FinsRPC: Finnish Prostate Cancer Screening Trial; HPFS: Health Professionals Follow-up Study; HOPE: Heart Outcomes Prevention Evaluation; HOPE-TOO: Heart Outcomes Prevention Evaluation–The Ongoing Outcomes; MEC: Multiethnic Cohort; NIH-AARP: National Institutes of Health-American Association of Retired Persons; NLCS: Netherlands Cohort Study; PHS II: Physicians’ Health Study II; PLCO: Prostate, Lung, Colorectal, and Ovarian Cancer; PSA: Prostate-specific antigen; RCT: Randomised controlled trial; SELECT: Selenium and Vitamin E Cancer Prevention Trial; SU.VI.MAX: Supplementation en Vitamines et Mineraux Antioxydants;VITAL: VITamin D and OmegA-3 TriaL; WNYDS: Western New York Diet Study.

**Table 2:** Subgroup analyses of studies on vitamin E intake and prostate cancer.

Type of Vitamin E Intake	No. of Studies	Sample Size	RR (95% CI)	I*^2^* Value (%)	*p* Value for Subgroup Differences
**Dietary intake**					
**Study design**					
Case-control studies	13	18,322	0.93 (0.84–1.02)	28.34	0.289
Cohort studies	6	498,431	0.99 (0.93–1.05)	12.48	
**Sample size**					
<1000	7	4087	0.77 (0.54–1.10)	72.12	0.605
>1000	12	512,666	0.98 (0.93–1.03)	5.22	
**Geographical region**					
North America	8	474,184	0.99 (0.93–1.06)	12.40	**0.036**
Europe	9	40,398	0.92 (0.84–1.01)	0.02	
**Vitamin E intake** ^a^					
≥15 mg/day	10	189,841 133,729	0.94 (0.86–1.02)	0.00	
**Supplemental intake of vitamin E**					
**Study type**					
Observational	12	613,469	0.99 (0.95–1.04)	0.04	0.133
Interventional	6	72,879	0.96 (0.82–1.13)	72.90	
**Study type**					
Case-control studies	3	7640	0.88 (0.63–1.22)	51.49	0.451
Cohort studies	9	605,829	1.00 (0.95–1.04)	0.00	
**Sample size**					
<20,000	10	67,433	1.00 (0.91–1.10)	34.36	0.094
>20,000	8	618,915	0.98 (0.94–1.03)	11.20	
**Geographical region**					
North America	13	609,392	1.01 (0.97–1.06)	18.76	**0.020**
Europe	4	69,960	**0.81 (0.69–0.97)**	33.56	
**Dose of supplements used**					
≥400 IU	7	457,383	1.00 (0.93–1.08)	40.67	
RCTs using dose <400 IU/day	3	48,147	0.85 (0.67–1.09)	69.43	0.541
RCTs using dose ≥400 IU/day	3	24,732	1.11 (0.97–1.27)	16.82	
**Study population**					
RCTs participants without underlying conditions	4	65,580	0.93 (0.74–1.18)	86.18	0.712
RCTs participants with underlying conditions	2	7299	0.99 (0.80–1.23)	0.00	

Abbreviations: IU, International units; RCT: Randomized controlled trial. ^a^ Recommended daily intake by the Institute of Medicine Panel on Dietary Antioxidants and Related Compounds in the United States. The guidelines for intake may differ from country to country and can vary from 3 mg/day to 15 mg/day [53]. Bolded values represent a statistically significant result at *p* value < 0.05.

## Data Availability

Data described in the manuscript, code book, and analytic code will be made available upon request from the corresponding author, pending application and approval.

## Supplementary Materials

The following supporting information can be downloaded at: https://www.mdpi.com/article/10.3390/nu15010014/s1, Table S1: Search terms used to identify relevant references.
