# Supplementary material for: Vitamin E Intake and Risk of Prostate Cancer: A Meta-Analysis
Source: Nutrients. 2022 Dec 21;15(1):14. doi: 10.3390/nu15010014 (PMC9824720; doi:10.3390/nu15010014)
Supplement: Supplementary file 1 [file nutrients-15-00014-s001.zip › nutrients-1942303-supplementary.pdf]

nutrients

Supplementary Materials

Vitamin E Intake and Risk of Prostate Cancer: A Meta-Analysis

Wei Qi Loh [1], Jiyoung Youn [1,2] and Wei Jie Seow [1,*]

[1] Saw Swee Hock School of Public Health, National University of Singapore and National University Health System, 12 Science Drive 2, #10-01, Singapore 117549, Singapore
[2] Department of Food and Nutrition, College of Human Ecology, Seoul National University, Seoul 08826, Republic of Korea
* Correspondence: ephswj@nus.edu.sg; Tel.: +65-6601-1243

Table S1. Search terms used to identify relevant references.

Database		Search strategy
PUBMED	#1	Vitamins [MeSH]
	#2	Vitamin*[Title/Abstract] OR micronutrient*[Title/Abstract] OR tocopherol*[Title/Abstract] OR tocotrienol*[Title/Abstract]
	#3	#1 OR #2
	#4	Prostatic Neoplasms[MeSH]
	#5	Prostate[Title/Abstract] OR prostatic[Title/Abstract]
	#6	neoplasm*[Title/Abstract] OR cancer*[Title/Abstract] OR tumor*[Title/Abstract] OR tumour*[Title/Abstract] OR carcinoma*[Title/Abstract] OR malignan*[Title/Abstract] OR adenocarcinoma*[Title/Abstract]
	#7	#5 AND #6
	#8	#4 OR #7
	#9	#3 AND #8
EMBASE	#1	'vitamin'/exp
	#2	vitamin*:ab,ti OR micronutrient*:ab,ti OR tocopherol*:ab,ti OR tocotrienol*:ab,ti
	#3	#1 OR #2
	#4	'prostate tumor'/exp
	#5	prostate:ab,ti OR prostatic:ab,ti
	#6	neoplasm*:ab,ti OR cancer*:ab,ti OR tumor*:ab,ti OR tumour*:ab,ti OR carcinoma*:ab,ti OR malignan*:ab,ti OR adenocarcinoma*:ab,ti
	#7	#5 AND #6
	#8	#7 OR #4
	#9	#3 AND #8
Cochrane Library	#1	vitamin[MeSH]
	#2	(vitamin* or micronutrient* or tocopherol* or tocotrienol*):ab,ti,kw
	#3	#1 OR #2
	#4	prostatic neoplasms[MeSH]
	#5	(prostate or prostatic):ti,ab,kw
	#6	(neoplasm* or cancer*or tumor* or tumour* or carcinoma* or malignan* or adenocarcinoma*):ti,ab,kw
	#7	#5 AND #6
	#8	#4 OR #7
	#9	#3 AND #8
